# The algorithm for Alzheimer risk assessment based on *APOE* promoter polymorphisms

**DOI:** 10.1186/s13195-016-0187-9

**Published:** 2016-05-19

**Authors:** Anna Limon-Sztencel, Beata S. Lipska-Ziętkiewicz, Magdalena Chmara, Bartosz Wasag, Leszek Bidzan, Beata R. Godlewska, Janusz Limon

**Affiliations:** Copernicus Hospital, Nowe Ogrody 1-6 Street, 80-803 Gdansk, Poland; Department of Biology and Genetics, Medical University of Gdansk, Debinki 1 Street, 80-210 Gdansk, Poland; Department of Developmental, Psychotic, and Geriatric Psychiatry, Medical University of Gdansk, Debinki 7 Street, 80-952 Gdansk, Poland; University Department of Psychiatry, University of Oxford, Oxford, OX3 7JX UK

**Keywords:** Alzheimer’s disease, *APOE*, *APOE* promoter polymorphisms, Apolipoprotein E isoforms, Risk factor

## Abstract

**Background:**

Over the past two decades, the *APOE* gene and its polymorphisms have been among the most studied risk factors of Alzheimer disease (AD) development; yet, there are discrepancies between various studies regarding their impact. For this reason, the evaluation of the *APOE* genotype has not been included in the current European Federation of Neurological Societies guidelines for AD diagnosis and management. This aim of this study was to add to this discussion by assessing the possible influence of multiple polymorphisms in the promoter region of the *APOE* gene and genotypes of its allele E on the risk for dementia.

**Methods:**

We performed a comprehensive analysis of *APOE* gene polymorphisms, assessed the detected genotypes and correlated molecular findings with serum apolipoprotein E concentrations. The study comprised 110 patients with AD and 110 age-matched healthy individuals from the Polish population.

**Results:**

Four polymorphisms of the *APOE* gene had minor allele frequency exceeding 5 % and were included in the analysis: −491A/T (rs449647), −427T/C (rs769446), −219T/G (rs405509) in the promoter region and +113G/C (rs440446) in intron 1. A protective effect of the −219G allele on AD development was observed. Also, the −491T and −219G alleles were found to be underrepresented in the carriers of the *APOE* E4 variant. On the basis of the genotype and linkage disequilibrium studies, a relative score was attributed to given genotypes with respect to the estimated probability of their protective effects against AD, giving rise to the ‘preventive score’. This ‘preventive score’, based on the total sums of the relative scores, expresses the protective effect deriving from the synergistic action of individual single-nucleotide polymorphisms. The ‘preventive score’ was identified as an independent predictive factor.

**Conclusions:**

We propose a novel, more complex approach to AD risk assessment based on the additive effect of multiple polymorphic *loci* within the *APOE* promoter region, which on their own may have too weak an impact to reach the level of significance. This has potentially practical implications, as it may help to improve the informative potential of *APOE* testing in a clinical setting. Subsequent studies of the proposed system in large, multi-ethnic cohorts are necessary for its validation and to assess its potential practical value for clinical applications.

**Electronic supplementary material:**

The online version of this article (doi:10.1186/s13195-016-0187-9) contains supplementary material, which is available to authorized users.

## Background

In various studies on multiple distinct ethnic groups and several different data sets performed over the last two decades, it has been shown that the epsilon 4 (E4 or ε4) variant of the apolipoprotein E (*APOE*) gene is associated with an increased risk for both sporadic and familial forms of Alzheimer disease (AD). However, it is also generally acknowledged that the *APOE* E4 variant alone is neither indispensable nor sufficient to cause the disease [1]. Subsequent series of genome-wide association studies performed with the aim of identifying further genetic predisposition sites produced contradictive outcomes [2]. Therefore, in the pursuit of identifying AD risk factors, additional *APOE* gene polymorphisms in the transcriptional regulatory regions of the gene —the −1000 to +400 proximal promoter region in particular—were investigated [1]. Three single-nucleotide polymorphisms (SNPs) were identified as the most promising: −491A/T (rs449647), −427T/C (rs769446) and −219T/G (rs405509) [3]. Of these, rs449647 AA and rs405509 TT genotypes were most commonly associated with AD. A number of validation studies, including a large meta-analysis consisting of 1732 patients with dementia and 1926 healthy control subjects [4], performed with individuals from various ethnic groups supported this association. The rs449647 polymorphism was shown to affect constitutional *APOE* transcriptional level in vitro [5], with its A allele found to increase *APOE* promoter activity and to confer an increased risk of AD independently of *APOE* E4. Alleles C and G of rs769446 and rs405509 polymorphisms, respectively, were also shown to increase *APOE* promoter activity [6, 7]. Further *in silico* studies provided additional evidence that these promoter polymorphisms are functional [1]. Interestingly, some studies suggested the role of rs405509 polymorphism to be age-dependent, with a more pronounced effect in the older population, both in the context of normal aging [8] and in the development of dementia [4, 9]. However, not all studies reproduced the reported associations and/or showed rs449647, rs769446 and rs405509 polymorphisms to be independent of E4 status [1, 9, 10]. In an attempt to clarify such discrepancies, our aim in this study was to assess possible associations between polymorphisms in the promoter region of the *APOE* gene and genotypes of its allele E, and the risk for dementia. We also aimed to assess associations of these polymorphisms with levels of the APOE protein in the serum. An additional aim was to assess the existence of the linkage (haplotype analysis) between *APOE* gene polymorphisms and dementia syndrome.

## Methods

### Study group

Our study included 110 patients with confirmed AD recruited from psychiatric hospitals and outpatient clinics by specialists in geriatric psychiatry. The diagnosis of AD was made on the basis of medical interviews, clinical symptoms and appropriate imaging examinations and clinical scales. All patients had a magnetic resonance imaging (MRI), computed tomography or single-photon emission computed tomography examination done and were tested on clinical scales and tests including the Hachinski Ischemic Scale, the Geriatric Depression Scale, the Mini Mental State Examination (MMSE) and the Clock Drawing Test. Relevant tests aimed at eliminating other possible causes of impairments in cognitive function were performed during the diagnostic process. These included a complete blood count, a lipidogram and other tests clinically appropriate for somatic diseases the patients had.

The age-matched control group consisted of adults (*n* = 110) with no signs or symptoms of dementia or a severe somatic disorder. In the whole group, a detailed questionnaire was completed by the physicians in charge, addressing putative environmental risk factors and/or the presence of somatic co-morbidities. All participants were of European origin and homogeneous ethnic (Polish) background.

### Molecular and biochemical studies

Genomic DNA was extracted from peripheral blood leucocytes using ionic detergent lysis and proteinase K digestion, phenol/chloroform extraction and isopropanol precipitation according to standard methods. *APOE* E2/E3/E4 alleles (rs429358, rs7412) and promoter polymorphisms (rs439382, rs1799981, rs1081103, rs72654465, rs449647, rs1799982, rs769446, rs72654466, rs405509, rs72654467, rs9282609, rs440446, rs877973, rs769447) were determined by polymerase chain reaction (PCR) followed by Sanger sequencing. The following pairs of primers were used: 5′-TCT TGC TGA GGC TGG AGT G-3′ and 5′-CAA GGA TCC CAG ACT TGT CC-3′, 5′-AAG ACC TCT ATG CCC CAC CT-3′ and 5′-CCA GTC TCG CAT TCC TCA TT-3′, 5′-ACG CGG GCA CGG CTG TCC AAG GAG-3′ and 5′-CTC GCG GGC CCC GGC CTG GTA CAC-3′. Bidirectional sequencing analysis of PCR products was performed using an Applied Biosystems 3130 Genetic Analyzer and the BigDye Terminator v3.1 Cycle Sequencing Kit (Applied Biosystems, Foster City, CA, USA). Sequences were analysed using Sequencher version 4.10.1 DNA sequencing software (Gene Codes Corporation, Ann Arbor, MI, USA). The ApoE level (expressed as milligrams per decilitre) was estimated by using the electroimmunodiffusion method using HYDRAGEL Protein(E) assay (Sebia, Evry Cedex, France) as described previously [11].

### Statistical analyses

Minor allele frequency (MAF) <5 % was the criterion for exclusion from further statistical analysis. Genotype analysis was performed using additive, genotypic and dominant models; (only significant findings are presented further in the paper). Frequencies were compared using χ^2^ tests with continuity corrections or Fisher’s exact test with Freeman-Halton extension for 2 × 3 and 2 × 4 tables when applicable. For continuous variables, differences between groups were evaluated using the Kruskal-Wallis test for global comparisons and Mann-Whitney *U* tests for pairwise comparisons. Unconditional logistic regression was used to examine the association between *APOE* genotypes and AD. In univariate analyses, the Bonferroni correction for multiple testing was applied. Background characteristics (age, sex, education level, residence) were incorporated into the analyses. In the logistic regression analysis, indicator variables for the sex, education level and residence were added to the models. All calculations were performed using STATISTICA 12 software (StatSoft, Tulsa, OK, USA) data analysis software system.

## Results

### Clinical and demographic data

No significant differences were noted between patients and control subjects with respect to age, family history or the most common factor risk factors associated with dementia: previous episodes of depression, past head traumas, concomitant cardiovascular disorders, diabetes or thyroid disease. The control group included more females due to availability of participants via established and ethically approved recruitment routes (see Table 1). The median MMSE scores were 20.3 (range 15–25) for the study group and 30 (range 28–30) for the control group.

**Table 1:** Clinical and demographic data for patients with Alzheimer disease and healthy volunteers

	Patients	Healthy volunteers
Sex, F:M	51:59	85:25
Age, years	71.2 (9, 48–89)	66.8 (7.5, 55–94)
Age at onset, years	67.9 (8.6, 46–85)	N/A
Time from disease onset, years	3.6 (1.9, 1–10)	N/A
Education level, *n*		
Higher education/university	25	64
Secondary education of general type	45	40
Secondary education of technical training type	18	4
Primary school education	22	0

*N/A* not applicable

Continuous variables are shown as mean (SD, range). There are no statistically significant differences between groups (*p* > 0.05) apart from sex distribution

### Univariable analyses of individual SNPs

The genotyping rate was 100 % in the study and control groups. Only six polymorphisms were found to have MAF exceeding 5 % and hence were eligible for statistical evaluation: rs449647, rs769446 and rs405509 in the promoter region; rs440446 in intron 1; and rs429358 and rs7412 in exon 4. The genotype distribution did not deviate significantly from the Hardy-Weinberg equilibrium. The observed frequencies of each SNP are presented in Table 2.

**Table 2:** Correlation between the studied SNPs of the *APOE* gene and the risk of AD, presence of *APOE* isoforms and ApoE serum levels

Genetic marker	MAF (patients with AD)	MAF (control group)	Alzheimer disease	Presence of E4 isoform	Presence of E2 isoform	ApoE serum level
rs449647 (AT)	15.0 %	21.8 %	Allele T OR 0.63 (95 % CI 0.39–1.01), *p* 0.07	Underrepresented in carriers of allele T^a,b^ genotypes AT, TT^a,b^	Correlates with allele T^a,c^ genotypes AT, TT^a,c^	Lower ApoE levels in carriers of allele A^d^
rs769446 (TC)	5.9 %	6.8 %	n.s.	n.s.	Correlates with allele C,^a,c^ genotypes TC, CC^a,c^	n.s.
rs405509 (TG)	39.8 %	55.9 %	Allele G^a,c^ OR 0.52 (95 % CI 0.36–0.75)	Underrepresented in carriers of allele G,^d^ genotypes TG, GG^a,b^	Correlates with allele G,^a,c^ genotypes TG, GG^a,c^	n.s.
rs440446 (CG)	33.5 %	38.6 %	n.s.	Underrepresented in carriers of allele C,^a,c^ genotypes GC, CC^a,c^	Correlates with allele C,^a,c^ genotypes GC, CC^a,c^	n.s.
rs429358 (TC)	28.9 %	9.1 %	Allele C^a,c^ OR 4.1 (95 % CI 2.4–6.9)	N/A	N/A	Lower ApoE levels in carriers of allele C,^a,b^ genotypes CT, CC^a,c^
rs7412 (CT)	3.9 %	6.4 %	n.s.	N/A	N/A	Higher ApoE levels in carriers of allele T^d^
E4	N/A	N/A	OR 4.7^a,c^ (95 % CI 2.5–8.6)	N/A	N/A	Lower ApoE^e^ levels in carriers of E4^a,c^
E2	N/A	N/A	OR 0.40 (95 % CI 0.15–1.07), *p* 0.1	N/A	N/A	Higher ApoE^e^ levels in carriers of E2^a,b^

*ApoE* apolipoprotein E, AD Alzheimer disease, *E* epsilon isoform of the apolipoprotein E, *MAF* minor allele frequency; *n.s.* not significant, *N/A* not applicable, *OR* odds ratio, *CI* confidence interval

^a^Associations that survived the correction for multiple testing (Bonferroni correction)

^b^ *p* < 0.01

^c^ *p* < 0.001

^d^ *p* < 0.05

^e^Detailed description with respect to all genotypes is provided in the Results section

For each of the studied SNPs of the promoter/intron 1 region, no correlations between age at disease onset, sex, family history or somatic co-morbidities were observed. The presence of allele G of rs405509 conferred a protective effect against AD (OR 0.52 (95 % CI 0.36–0.75; *p* < 0.001). A similar tendency was observed with respect to allele T of rs449647; however, the correlation did not reach the level of significance (Table 2). Alleles T, G and C of rs449647, rs405509 and rs440446 SNPs, respectively, were significantly underrepresented in the carriers of the E4 variant.

ApoE serum levels were significantly lower in patients with AD (5.7 mg/dl vs. 6.5 mg/dl; *p* < 0.0001). Of the studied SNPs, only the rs449647 polymorphism significantly correlated with ApoE serum concentration. The presence of its allele A was associated with lower ApoE levels. Also, the differences in ApoE levels with respect to *APOE* epsilon variants were statistically significant (*p* < 0.001). The lowest levels of ApoE were noted among E4 carriers (3.72 ± 0.8 mg/dl for E4/E4 genotype, 5.54 ± 1.2 mg/dl for E3/E4 genotype), while the highest values were observed in E2 carriers (7.04 ± 1.36 mg/dl for E2/E3 genotype, 7.23 ± 0.5 mg/dl for E2/E4 genotype). Cases with E3/E3 genotype had a mean value of 6.38 ± 1.48 mg/dl. No subject with E2/E2 genotype was present in either the study or the control group. Table 2 summarizes the results of univariable analyses.

### Haplotype and linkage disequilibrium analyses

Haplotype analysis of the entire *APOE* gene sequence (chromosome 19q, 50,100,879–50,104,489; *GRCh36.3/hg18*) was performed using Haploview 4.2 software, and data representative of the four distinct populations with African, Asian and European ancestry were retrieved from the HapMap database (http://hapmap.ncbi.nlm.nih.gov [accessed May 2011]) [12]. The analysis revealed five tag SNPs forming no haplotype blocks (Additional file 1). Subsequently, we evaluated the impact of *APOE* genotypes reported previously as predictive of the risk of AD [13]. In the genotypes comprising the E2 variant, no statistically significant differences between the study and control groups were observed. In the genotypes comprising at least one E4 variant, the −491AA/−219TT/E4 genotype was shown to increase the risk of AD (OR 5.4, 95 % CI 1.14–25.6; *p* = 0.02).

Afterwards, the experimental data for Polish population was analysed for the putative linkage disequilibrium (LD). LD between *loci* rs449647, rs405509 and rs440446 on one side, and the *APOE* exon 4 *loci* coding for epsilon variants (distant by 3 kb) on the other, was observed. Of all SNPs analysed, the strongest LD was observed for rs440446 and rs429358 (Lewontin coefficient D′ 0.956, logarithm of [base 10] odds [LOD] 11.51, *r*^2^ = 0.127). The rs440446 *locus* was simultaneously linked with promoter SNPs, too, for instance with rs405509 *locus* distant by 0.3 kb (Lewontin coefficient D′ 0.925, LOD 32.78, *r*^2^ = 0.428).

### Multivariable analyses of the analysed risk factors

In the multivariate logistic regression analysis, the initial testing was performed using six covariates identified in the aforesaid univariate analyses (−491A/T, −219 T/G, +113G/C, E4, E2, ApoE level) and the demographic indicator variables for sex, education level and residence. The initial analysis of all nine plausible factors identified four covariates: E4, education level, male sex and residence in large cities as the significant predictors of risk of AD development. Next, the best subsets search method was used to verify the optimal set of predictors. The model likelihood statistics were computed for every possible predictor subset to identify the best subset. Eventually, the best model comprising four independent predictors of AD development was identified. The significant factors included two associated with higher risk (E4 with OR 4.12, 95 % CI 1.55–11.00; male sex with OR 3.26, 95 % CI 1.46–7.27) and two associated with lower risk (residence in large cities with OR 0.04, 95 % CI 0.01–0.37; education level with OR 0.23, 95 % CI 0.14–0.40).

### ‘Preventive score’

The results of simple and multivariable analyses of promoter SNPs along with linkage disequilibrium data were ultimately used as the foundation for the ‘preventive score’. Since, on the one hand, none of the studied promoter SNPs was identified as an independent prognostic factor by logistic regression, and on the other, the promoter SNPs were found in strong linkage disequilibrium, we decided to test the plausible additive benefit of their joint analysis. Accordingly, for a given genotype, a relative score was attributed with respect to its estimated probability of protective effect against AD (Table 3).

**Table 3:** Relative scores for individual *APOE* promoter single-nucleotide polymorphism genotypes used for evaluation of the ‘preventive score’ against Alzheimer disease

Genotype	Relative score
rs449647: AT heterozygote	1
rs449647: TT homozygote	2
rs405509: TG heterozygote	1
rs405509: GG homozygote	2
Other genotypes	0

The risk for AD development expressed as the ‘preventive score’ resulting from the total sum of the relative scores (minimum 0, maximum 4) was found to be inversely related to its value. The average ‘preventive score’ in the AD group was 1.08, compared with 1.55 in the control group (*p* = 0.0001). ‘Preventive score’ correlated with presence of the E2 allele (Spearman rank correlation 0.40; *p* < 0.05) and inversely with the E4 allele (Spearman rank correlation 0.13; *p* < 0.05). The ‘preventive score’ correlated significantly with protein ApoE levels (Spearman rank correlation 0.25; *p* < 0.05), while such a correlation was not seen for individual SNPs.

The significance of 'preventive score' was further tested with multivariate logistic regression, performed in the same way as described above. The only difference was the replacement of the −491A/T, −219T/G, +113G/C and E2 covariates by the ‘preventive score’. The resulting best subset consisted of five independent prognostic factors: two associated with higher risk (E4 with OR 3.96, 95 % CI 1.90–8.26; male sex with OR 3.09, 95 % CI 1.42–6.74) and three associated with lower risk (education level with OR 0.25, 95 % CI 0.15–0.41; residence in large cities with OR 0.04, 95 % CI 0.01–0.38; ‘preventive score’ with OR 0.62, 95 % CI 0.40–0.96). The additive benefit of the ‘preventive score’ is roughly half the effect of E4 in the model.

Additional statistical analyses using the logistic regression model in the subgroups of patients resulting from an inclusion/exclusion of the E2 and E4 allele carriers and in the sub-group homozygous for the E3 allele, have shown that the ‘preventive score’ allows for further delineation of the eventual risk of AD development in the subgroup of E4 carriers (for more details, see Additional file 2).

## Discussion

We report a 9.1 % incidence of the *APOE* E4 variant in our sample derived from the Polish population, which is in line with previously published values [14]. Similarly to previous studies, we were able to show that the presence of at least one E4 variant confers almost a five times higher risk of AD development and that this correlation is gender-dependent [2]. For women, the presence of E4 variant conferred almost an eight times higher risk, while for men the association was not statistically significant (data not shown). In contrast to previously published results, no correlation between the presence of the E4 variant and the age of AD onset was observed [15]. However, our study group did not include many individuals diagnosed when they were older than 80 years of age, as late-onset AD was not a priority in our study. This might have resulted in the lack of such a correlation in our data.

Despite the reproducibility of the *APOE* E4 variant association with AD development, also shown in our study, the pathomechanism of such a correlation remains largely unsolved. Hence, an intense search for additional modifying factors, both intragenic and located at other *loci* of the genome, has been conducted worldwide. Of these, two *APOE* promoter SNPs—rs449647 (−491A/T) and rs405509 (−219T/G)—were shown to have predictive value for AD development [1–3, 5, 6, 9, 16–22]. However, not all studies were able to reproduce the protective effect in their populations, for instance in French [10], Irish [23], Finnish [24] or Japanese [25]. Hence, the exact role of these SNPs and their interaction with epsilon variants—in other words, their predictive character being independent or derivative of epsilon status—remains a subject of intensive debate.

In the present study, we were able to show a protective effect of −219G (rs405509) allele in a sample derived from the Polish population, while the correlation with the −491T (rs449647) allele did not reach the level of significance. This is interesting in light of an MRI study by Chen et al. [4], who showed an accelerated age-related reduction of thickness in the left parahippocampal gyrus in −219 TT carriers and suggested it as the neural substrate underlying a faster decline in cognition in individuals with this genotype. Hence, carrying a G allele might offer protection against such a process and act protectively in terms of cognition. The Chen et al. [4] study, as well as a few others (see, e.g., [9]), showed the effect of this polymorphism to be age-related. As most individuals (95 of 110) in our study were mostly older than 60 years of age, this effect might have been more pronounced.

In addition, we observed that both alleles, −219G and −491T, were significantly underrepresented in the carriers of the E4 variant. Such characteristics render the variants plausible candidates for independent risk/protective factors for AD development. However, the latter was not confirmed in the multivariable logistic regression analysis.

The inconsistent findings in previous studies regarding the presence of LD between promoter polymorphisms and the *APOE* epsilon variant coding SNPs were explained by the suggestion that LD may vary significantly depending on ethnic background. Here, we have shown the presence of a significant LD in the coherent group of Polish patients, being a large ethnic group with a low rate of consanguinity. The analysis of data retrieved from the HapMap project showed no haplotype blocks within the *APOE* gene. However, various differences in LD between individual SNPs have been observed in all ethnic groups. Lescai et al. [13] performed a similar, large-scale haplotype analysis in a group of more than 1000 Italian subjects and showed the presence of individual LDs but not haplotype blocks. In their study of the prognostic role of *APOE* SNPs on AD risk, the only significant additive effect has been observed for the rs405509 allele T/E4 haplotype present in phase (i.e., ‘*in cis*’). They did not perform a more comprehensive analysis that would include other in phase SNP *loci*. In our present study, we were able to show that the presence of the −491AA/−219TT/E4 genotype appears to be even more informative. It has been shown that lower ApoE levels are associated with a higher risk of developing AD. As −491A, −219T and E4 alleles have all been shown to independently decrease *APOE* gene expression, and hence ApoE levels [5–7], a haplotype containing all three alleles might confer a higher risk for AD because of the synergistic action of all three variants. Promoter SNPs confer a mild but additive effect on *APOE* expression. We showed that the ‘preventive score’ correlated significantly with protein ApoE levels, while this was not the case when individual SNPs were considered. It is possible that the SNPs need to be assessed jointly for their impact to reach significance, which might be one of the possible explanations for discrepancies in the previous reports. The results of the analysis of the above-tested polymorphisms in the promoter region of the *APOE* gene, in addition to increasing knowledge of the allelic variants of the gene, can be important for determining the risk of the incidence of dementia.

We believe that one of the most important findings of this study is the identification of the ‘preventive score’. Its importance derives from the fact that it takes into account an additive risk related to the presence of alleles and genotypes, which on their own may have too weak an impact to reach the level of significance. This has potentially practical implications, as it may help improve the informative potential of *APOE* testing in a clinical setting. The *APOE* testing, limited to the evaluation of the E4 variant, was not found to be sufficiently informative to be included in the current European Federation of Neurological Societies guidelines for AD diagnosis and management [26]. Here, we show that the ‘preventive score’ has an independent prognostic value, regardless of E4 status. Moreover, it has been shown to be more informative than serum ApoE levels, the ultimate marker of gene expression. The latter, owing to the complexity of protein-protein interactions and the impossibility of their direct measurement in the tissue of choice (i.e., brain), appears to be a less preferable parameter.

One important issue is the relationship between the ‘preventive score’ and epsilon variants, namely whether the ‘preventive score’ is independent of their effect, and especially the effect of the E2 allele. We attempted to tackle this issue by performing additional statistical analyses using the logistic regression model in the subgroups of patients resulting from inclusion/exclusion of the E2 and E4 allele carriers and in the subgroup homozygous for the E3 allele. They have shown that the ‘preventive score’ allows for further delineation of the eventual risk of AD development in the subgroup of E4 carriers and supports the role of the ‘preventive score’ as an additional diagnostic tool. However, these results need to be viewed with caution, as the sizes of the subgroups after the removal of the E2 and E4 alleles carriers were small (for example, only 5 % of patients with AD and 12 % of control subjects were E2 carriers), and a well-discriminating distribution of the epsilon alleles was not possible to obtain; for example, E2 homozygotes were lacking in both cohorts.

We believe that the utility of the ‘preventive score’ is an interesting and potentially useful finding; however, our results need to be replicated to confirm the validity of the score. Hence, future studies by independent research groups with larger numbers of patients are warranted.

## Conclusions

We propose the ‘preventive score’ as a new predictive factor in AD. In contrast to known AD susceptibility markers, the score does not predispose to AD development but allows an estimation of individual risk for the disease. Nevertheless, further studies with large, multi-ethnic cohorts are necessary to establish its realistic utility in clinical settings.

## Ethical approval and consent to participate

All patients and healthy individuals gave written informed consent for molecular genetic testing. The study was approved by the Independent Bioethics Commission for Research at the Medical University of Gdansk. The experiments were done in accordance with the Helsinki Declaration of 1975.

## Additional files

Additional file 1: Haplotype analysis of the SNPs representative of *APOE locus*. Results of the haplotype analysis of the SNPs representative of *APOE* *locus* based on data retrieved from the HapMap Project database. (PDF 348 kb) Additional file 2: Logistic regression analyses in the subgroups of patients carrying E2/E3/E4 alleles. Summary of the statistical analyses using the logistic regression model approach in the subgroups of patients resulting from inclusion and/or exclusion of the E2 and E4 allele carriers and in the subgroup homozygous for the E3 allele. (PDF 312 kb)

## Abbreviations

AD: Alzheimer disease
ApoE: apolipoprotein E
E: epsilon isoform of the apolipoprotein E
LD: linkage disequilibrium
LOD: logarithm of (base 10) odds
MAF: minor allele frequency
MMSE: Mini Mental State Examination
MRI: magnetic resonance imaging
PCR: polymerase chain reaction
SNP: single-nucleotide polymorphism
