# Supplementary material for: The algorithm for Alzheimer risk assessment based on APOE promoter polymorphisms
Source: Alzheimers Res Ther. 2016 May 19;8:19. doi: 10.1186/s13195-016-0187-9 (PMC4872351; doi:10.1186/s13195-016-0187-9)
Supplement: Additional file 1: — Haplotype analysis of the SNPs representative of APOE locus. Results of the haplotype analysis of the SNPs representative of APOE locus based on data retrieved from the HapMap Project database. (PDF 348 kb) [file 13195_2016_187_MOESM1_ESM.pdf]

Additional file 1.

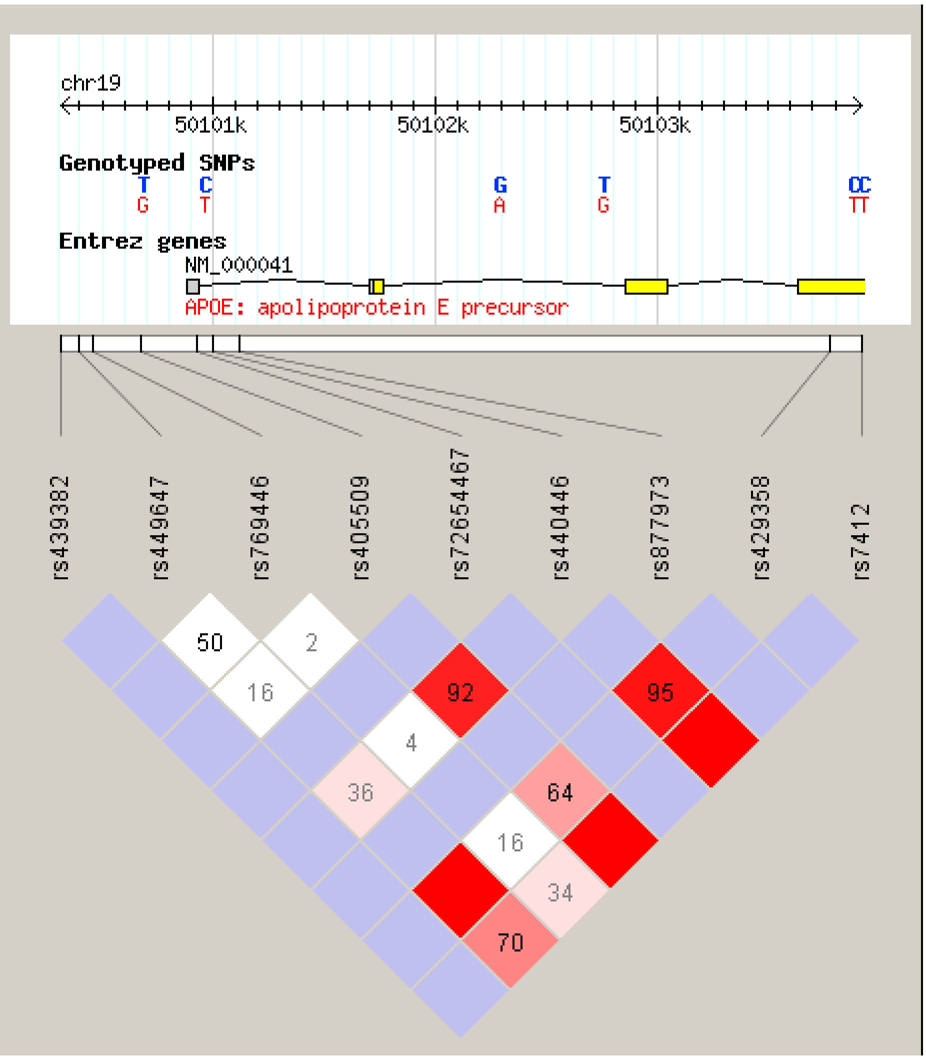

Haplotype analysis of the SNPs representative for *APOE locus*.

Results of the haplotype analysis of the SNPs representative for *APOE* locus based on the data retrieved from the HapMap Project database. The analysis was performed using Haploview software [10]. In the upper part of the scheme physical map of the corresponding fragment of the chromosome 19 is presented, the middle section shows exact intronic/exonic localization of the genotyped SNPs and the lower part illustrates identified associations.

Color code reflects the strength of association:

WHITE COLOR: D' < 1, LOD<2 - insignificant;

BLUE COLOR: D' = 1 LOD<2 high degree of recombination;

INTENSIVITY OF THE RED COLOR: D' < 1; LOD = 2 reflects the strength of association, the maximum of intensity is reached at D' = 1, LOD = 2 – high level of association. The numbers on the diamonds show D' value multipliedby 100.
