# Supplementary material for: The algorithm for Alzheimer risk assessment based on APOE promoter polymorphisms
Source: Alzheimers Res Ther. 2016 May 19;8:19. doi: 10.1186/s13195-016-0187-9 (PMC4872351; doi:10.1186/s13195-016-0187-9)
Supplement: Additional file 2: — Logistic regression analyses in the subgroups of patients carrying E2/E3/E4 alleles. Summary of the statistical analyses using the logistic regression model approach in the subgroups of patients resulting from inclusion and/or exclusion of the E2 and E4 allele carriers and in the subgroup homozygous for the E3 allele. (PDF 312 kb) [file 13195_2016_187_MOESM2_ESM.pdf]

Additional File 2. **Summary of the statistical analyses using the logistic regression model approach in the subgroups of patients resulting from an inclusion/exclusion of the E2 and E4 allele carriers and in the subgroup of homozygotes for the E3 allele.**

For detailed description of the statistical methods used see the main text.

1. The logistic regression in the subgroup of E2 carriers was impossible to be analyzed due to low number of subjects (n=6 AD, n=14 controls).

2. The logistic regression in the subgroup after exclusion of E2 carriers (n=104 with AD vs. n=96 controls) revealed that presence of E4 and male gender are independent risk factors while higher education and inhabitation in large cities are independent protective factors. In the same analysis, the 'preventive score' and APOE serum level were found insignificant and were excluded from the final best model (model likelihood statistics was computed for every possible predictor subset to identify the best subset).

3. The logistic regression in the subgroup after exclusion of E4 carriers (n=54 AD, n=90 controls) revealed that male gender is an independent risk factor while higher education, presence of E2 and inhabitation in large cities are independent protective factors. In the same analysis, the 'preventive score'and APOE serum level were found insignificant and were excluded from the final best model (model likelihood statistics was computed for every possible predictor subset to identify the best subset).

4. The logistic regression in the subgroup of E4 carriers (n=56 AD, n=20 controls) only two factors were identified as significant independent prognostic factors: 'preventive score' at OR=0.34 (95%CI 0.13-0.89; p=0.03) and higher education OR=0.35 (95%CI 0.13-0.94; p=0.04). Here, presence of E2, APOE serum level as well as other demographic factors were not selected to the best model (model likelihood statistics was computed for every possible predictor subset to identify the best subset). Yet, due to small number of cases, the obtained matrix included a "zero" call and hence its solution might be equivocal.

5. The logistic regression in the subgroup of E3/E3 individuals (n=51 AD, n=79 controls) revealed that male gender is an independent risk factor while higher education and inhabitation in large cities are independent protective factors. In the same analysis, the 'preventive score' and APOE serum level were found insignificant and were excluded from the final best model (model likelihood statistics was computed for every possible predictor subset to identify the best subset).
